# Focal Segmental Glomerulosclerosis: Genetics, Mechanism, and Therapies

**DOI:** 10.1155/2016/9643785

**Published:** 2016-06-26

**Authors:** Andreas Kronbichler, Jun Oh, Björn Meijers, Jae Il Shin

**Affiliations:** ^1^Department of Internal Medicine IV (Nephrology and Hypertension), Medical University Innsbruck, Anichstraße 35, 6020 Innsbruck, Austria; ^2^Pediatric Nephrology, University Medical Center Hamburg-Eppendorf, 20246 Hamburg, Germany; ^3^Department of Microbiology and Immunology, KU Leuven and Department of Nephrology, UZ Leuven, 3000 Leuven, Belgium; ^4^Department of Pediatric Nephrology, Severance Children's Hospital, Yonsei University College of Medicine, Seoul 120-752, Republic of Korea

Focal segmental glomerulosclerosis (FSGS) is one of the primary glomerular disorders in both children and adults which can progress to end-stage renal failure. Recent advances in cell biology and genetics have found new molecules and signaling mechanisms in podocytes for the pathogenesis of FSGS. In addition, evidence-based approaches will guide to the best and appropriate therapeutic options both in children and in adults with FSGS in this special issue.

The exact steps in the etiopathogenesis of FSGS are incompletely understood. In an attempt to summarize findings related to the immunologic changes, A. Kronbichler et al. provide an overview of T-cell, CD80 (B7-1), complement, chemokines/cytokines, macrophages, and B-cells in FSGS. Moreover, the authors highlight a potential transition into current and future potential therapeutic options. E. Königshausen and L. Sellin reviewed potential circulating permeability factors in primary FSGS that have been implicated in the pathogenesis due to the potential recurrence in renal allografts after kidney transplantation (KT), focusing on the soluble urokinase plasminogen activator receptor (suPAR), cardiotrophin-like cytokine factor-1 (CLCF-1), and CD40 antibodies. Secondary FSGS is an emerging cause of progressive kidney function decline. With the increased understanding of podocyte biology, several steps have been identified leading to podocyte injury and eventually glomerulosclerosis. In an elegant overview, J. S. Kim et al. provide an overview of common causes leading to secondary FSGS, including adaptive (with reduced or normal renal mass), drug-induced, genetic, or infection-related disease forms.

Regarding pathologic classifications of FSGS, M.-H. Han and Y.-J. Kim reviewed the Columbia classification, which distinguishes five variants (collapsing, tip, cellular, perihilar, and not otherwise specified) and has been widely used over the past 10 years, and pointed out the confusion about terminology of variants and difficulty in its application and the interpretation of lesions with mixed features in the same tissue specimen or evolution of the lesions. For the diagnosis of FSGS, B. Sprangers et al. reviewed on the diagnostic approaches and work-up to the patients using the potential biomarkers of FSGS, genetic testing, and histology.

Treatment of primary FSGS remains a challenge for the treating physicians, since repeated doses of long-term steroid use and steroid-dependence and steroid-resistance are leading to significant morbidities in both children and adults. K. H. Han and S. H. Kim reviewed on the current updated strategies for treatment of primary FSGS in children, including traditional therapies consisting of corticosteroids and calcineurin inhibitors and novel therapies such as rituximab, abatacept, adalimumab, and fresolimumab. A. Beer et al. focused on studies reporting on treatment outcome published during the last two decades. Unfortunately, there has not been a major progress in the treatment of adults and novel substances, such as sirolimus; adalimumab or galactose should not be considered as effective immunosuppressive measures. While rituximab, mycophenolate mofetil, or calcineurin inhibitors may be used in steroid-dependence, evidence is limited for the former two substances in resistant cases and other strategies such as extracorporeal treatment (immunoadsorption or plasma exchange) and alkylating agents may be used.

FSGS is known to recur after KT and is an important cause of graft loss early after transplantation. H. G. Kang et al. reviewed on the risk factors of FSGS recurrence after KT in children, potential biomarkers for predicting recurrence, treatment of FSGS recurrence after KT, and the strategies to prevent recurrences. M. Rudnicki reviewed on FSGS recurrence in adults after KT, emphasizing prophylactic and perioperative treatment with plasmapheresis and high-dose (intravenous) cyclosporine as the main cornerstones of immunosuppressive therapy and recent promising results of rituximab therapy.

Articles published in this special issue covered the various fields of FSGS such as pathophysiologic mechanisms of primary and secondary FSGS, histopathologic considerations, potential biomarkers and diagnostic work-up, evidence-based therapeutic strategies, and recurrence of FSGS after KT. Understanding theses various faces of FSGS will lead to better patient treatment and outcome.

*Andreas Kronbichler* *Jun Oh* *Björn Meijers* *Jae Il Shin*

